# Carrier-induced transient defect mechanism for non-radiative recombination in InGaN light-emitting devices

**DOI:** 10.1038/srep24404

**Published:** 2016-04-14

**Authors:** Junhyeok Bang, Y. Y. Sun, Jung-Hoon Song, S. B. Zhang

**Affiliations:** 1 Department of Physics, Applied Physics & Astronomy, Rensselaer Polytechnic Institute, Troy, NY 12180, USA; 2 Spin Engineering Physics Team, Korea Basic Science Institute (KBSI), Daejeon 305-806, South Korea; 3 182 Shingwan, Department of Physics, Kongju National University, Kongju Chungnam 314-701, South Korea

## Abstract

Non-radiative recombination (NRR) of excited carriers poses a serious challenge to optoelectronic device efficiency. Understanding the mechanism is thus crucial to defect physics and technological applications. Here, by using first-principles calculations, we propose a new NRR mechanism, where excited carriers recombine via a Frenkel-pair (FP) defect formation. While in the ground state the FP is high in energy and is unlikely to form, in the electronic excited states its formation is enabled by a strong electron-phonon coupling of the excited carriers. This NRR mechanism is expected to be general for wide-gap semiconductors, rather than being limited to InGaN-based light emitting devices.

Nonradiative recombination (NRR) refers to physical processes in semiconductors under electrical or optical excitations, where electrons and holes recombine without emitting photons. NRR is currently the most important factor limiting the efficiency of optoelectronic and photovoltaic devices in energy applications^1^,^2^. A good example is the efficiency loss in white light-emitting diodes (LEDs) based on GaN and its alloys^3^,^4^,^5^,^6^,^7^,^8^,^9^,^10^. The white LEDs hold great promises to revolutionize current lighting technology^11^,^12^. Their efficiencies, however, are still not enough to penetrate the general lighting market, which is currently dominated by cheep compact fluorescent lamps. Revealing the physics of NRR in such devices is therefore critical to fostering new technology breakthroughs.

The field of NRR study is dominated by the widely-accepted Shockley-Read-Hall (SRH)^13^,^14^ model and the Auger recombination (AR) model^1^,^4^,^5^,^6^. In the SRH model, defect with deep levels inside the band gap assist carrier recombination such that the energy of the excited carriers is dissipated through lattice vibration or phonon emission. In recent years, other defect-specific NRR processes have also been proposed^15^,^16^. In the AR process, in contrast, the carrier recombination is mediated by carrier-carrier scattering and the energy is transferred by generating higher energy carriers inside bulk energy continuum, which is then dissipated through phonon emission. The two models can be characterized as a defect centric model and a defect-free model, which has been the paradigm for NRR study over decades.

In this work, we show that the formation of defects, especially the Frenkel-pair (FP) defects, due to the presence of excited carriers creates a new type of NRR centers. The energy of the carriers is dissipated through a transient defect generation and annihilation process. In other words, it starts and ends with no deep level inside the band gap as opposed to the SRH mechanism, but the involvement of the transient defects makes it fundamentally different from the AR. Using first-principles calculations we found that in InGaN, the carrier-induced transient FP formation and associated NRR process can readily take place and compete effectively with radiative recombination. The transient nature of the NRR defects may have made them escape experimental detection, which is largely framed by the current thinking and lack of sub-ns-to-ns time resolutions.

To be more specific, first let us discuss the concept behind the carrier-induced transient-defect NRR. In a typical binary semiconductor, such as GaN, the top part of the valence band (VB) mainly consists of anion-derived bonding states, while the bottom part of the conduction band (CB) mainly consists of cation-derived anti-bonding states^17^,^18^. Figure 1(a) shows the initial occupations of the electronic states under carrier injection, e.g., in the active region of a working LED, where electrons and holes establish their respective quasi-equilibria at the VB and CB. When an anion is displaced from its original lattice site to an interstitial site, a Frenkel pair (FP) defect (i.e. a pair of anion vacancy and anion interstitial) is created. As the bonds between the anion and cation are broken, an anion-derived bonding state (near the interstitial) and a cation-derived anti-bonding state (near the vacancy) evolve from the VB and CB into the band gap as the interstitial and vacancy levels, respectively, as shown in Fig. 1(b). The interstitial and vacancy levels carry two holes and two electrons, respectively. Once the two levels approach each other inside the band gap, they exchange carriers. After the exchange, the interstitial moves back to annihilate the vacancy and heal the lattice, by which an NRR process is completed [see Fig. 1(c)].

In the ground state, the FP formation is prohibited because it would require a significant amount of energy to break the bonds and to lift up the (doubly-occupied) interstitial level. With excited carriers, however, such a process becomes possible^19^ because 1) the electrons in the CB will drop into the vacancy level, which further shifts downward due to FP formation, and 2) the holes in the VB will rise into the interstitial level, which further shifts up due to FP formation. Thus, both excited electrons and holes can offset the energy required in the FP formation process.

## Results

### Frenkel pair is unstable in ground state

As an alloy, InGaN can have different local structures made of In clusters (with up to four In atoms around a N atom). Since In-N bond is weaker than Ga-N bond, here for simplicity, we consider only the FP formation at a four In cluster (In_4_). This is reasonable because from the calculated formation energy of 90 meV, In_4_ concentration at a growth temperature of about 750 °C is on the order of 1.6 × 10^18 ^cm^−3^ for In_0.1_Ga_0.9_N samples. Figure 2 shows the atomic structures before and after the FP formation. The FP is formed by displacing the N at center to a neighboring interstitial site, which leaves an N vacancy (*V*_N_) behind. Figure 2(b) shows that the displaced N forms a split-interstitial with a neighboring N atom. This is consistent with the fact that, among isolated N interstitial configurations in GaN, split-interstitial is most stable^20^,^21^. The calculated FP formation energy is 4.42 eV in the electronic ground state, indicating that this is a forbidden process at room temperature. It also suggests that any FP generated during the NRR process will eventually recombine.

### Carrier relaxation in the NRR processes

Figure 3, light gray lines, shows the change of the energy levels during the FP formation assisted by charge carrier injection resulting in excited electrons near the CBM and excited holes near the VBM. One may notice from the figure that, even in defect-free InGaN, band edge energies deviate from their original positions due to occupation of excited states. This unphysical result is a drawback of the hybrid functional methods^22^,^23^,^24^. The errors have been corrected, according to the degree of localization of the states, yielding the red and blue lines in Fig. 3. It shows that when the N is displaced towards the interstitial site, the In-derived *V*_N_ level (which can host two electrons) decreases and the N-derived N interstitial (N_*i*_) level (which can host two holes) increases. Insets **a** and **b** in Fig. 3 show the charge density in the seventh configuration for *V*_N_ and N_*i*_, respectively, from which we can also see that the *V*_N_ gap state, evolved from the CB, has an anti-bonding character in regarding the broken bonds between the In atoms of the vacancy and the displaced N_*i*_. In contrast, the N_*i*_ gap state, evolved from the VB, has a bonding character between these states.

**Figure 1:**
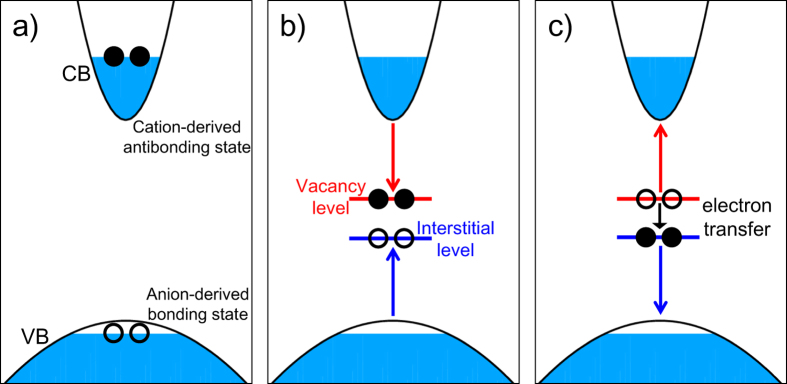
Schematic illustration of the change of the energy levels during NRR. (**a**) Initial defect-free state with injected carriers, (**b**) during FP formation, and (**c**) re-healing process back to defect-free ground-state after two-electron transfer. VB and CB denote the valence and conduction bands, respectively. Shaded blue regions represent occupied states. During the FP formation, an anion vacancy level and an anion interstitial level appear inside the band gap. The injected electrons (filled dot) at the highest occupied state in the CB drop to the vacancy level, whereas the injected holes (empty dot) in the VB raise to the interstitial level. After the electron transfer between the vacancy and interstitial levels, these levels undergo a reverse process to retreat back into the VB and CB, respectively.

**Figure 2:**
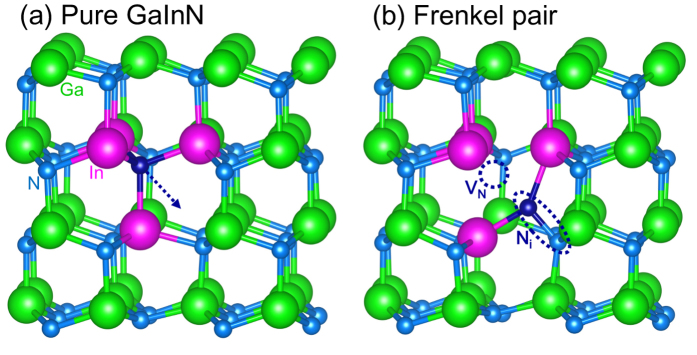
Atomic structure of (**a**) Pure InGaN and (**b**) with one FP defect. The moving N atom is denoted by a dark blue ball. In (**a**), arrow is the direction of the N displacement during the FP formation. In (**b**), split-interstitial and *V*_N_ are marked by dotted ellipse and circle, respectively.

**Figure 3:**
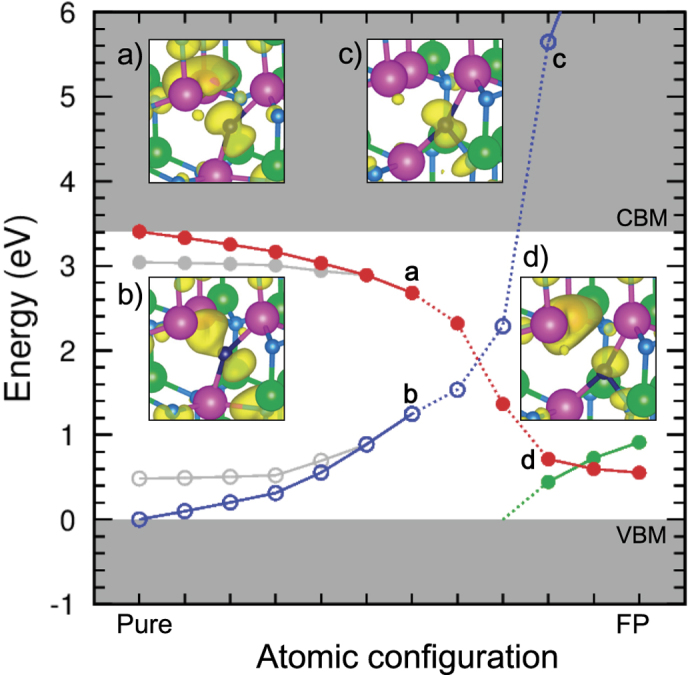
Change of energy level during FP formation with injected carriers. *V*_N_ and N_*i*_ levels are represented by red and blue lines, respectively. The filled (open) dot denotes fully occupied (empty) state. Dashed lines indicate the crossing region (explained in the text). Green line, popping up from VBM into the band gap, is another occupied N_*i*_ level. Insets are the charge density plots for the levels marked by a, b, c, and d. Gray lines are the uncorrected hybrid functional results.

Once the FP is formed (i.e., the final configuration in Fig. 3), the *V*_N_ level is further decreased to 0.55 eV above the VBM, whereas the N_*i*_ level is increased to 3.60 eV above the CBM. This implies that the levels have crossed due to the FP formation. In the crossing region (i.e., the eighth and ninth configurations in Fig. 3), however, it happens that the constrained self-consistent field (SCF) calculation dose not converge. Nevertheless, we can see the level crossing indirectly by checking the charge densities at the tenth configuration, as shown in the insets in Fig. 3. In contrast to insets **a** and **b** where the lower-energy state has an N lone pair, insets **c** and **d** show that the lower-energy state has In dangling bonds, instead. Note that, for clarity, the issue of level crossing was not included in in Fig. 1, as this schematic figure depicts isolated defects for which the cation level is higher than the anion level.

The lack of convergence in the crossing region can be attributed to charge sloshing between the N_*i*_ and *V*_N_ states at each SCF step. A similar situation takes place in conventional DFT calculations, when two or more partially occupied levels are nearly degenerate. One may overcome the problem by smearing the occupation over a certain energy range. Here, before entering the crossing region, two electrons occupy the high-lying *V*_N_ level and two holes occupy the low-lying N_*i*_ level. After exiting the crossing region, the system is electronically de-excited, so no empty state is below occupied states. To assess the effect of this occupation change, we resort to fractional occupation, in particular, for the two interacting levels, labeled *e*_*L*_ and *e*_*H*_, the following occupation numbers (*f*_*L*_, *f*_*H*_) = (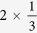, 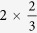) at the eighth configuration, and (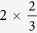, 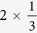) at the ninth configuration, are used. As expected, converged SCF results are obtained. To connect with the *V*_N_ and N_*i*_ levels in the non-crossing regions in Fig. 3, we define an average energy 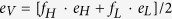 for occupied *V*_N_ and 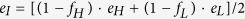 for empty N_*i*_. This yields smoothly connected energy levels between the excited and ground-state systems.

### Potential energy curves in the NRR process

**Figure 4:**
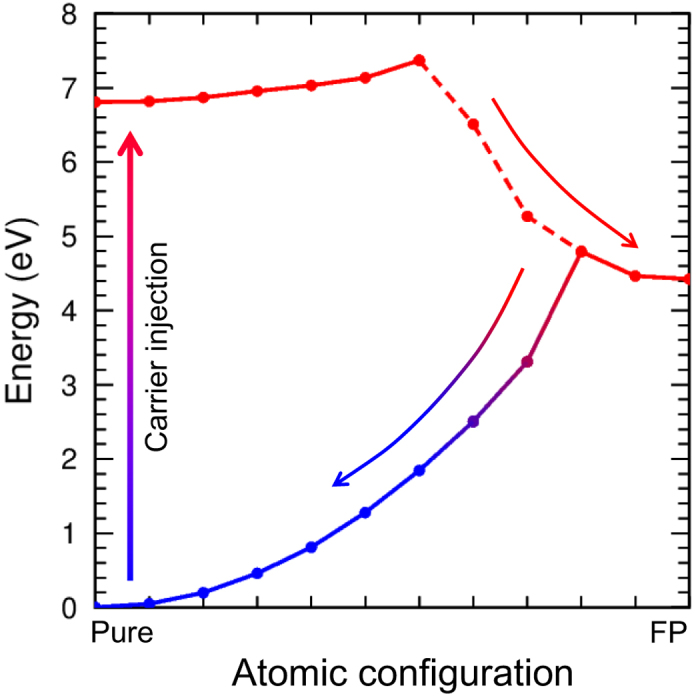
Potential energy curve in the NRR process. The FP formation and annihilation processes are corresponded to upper and lower lines, respectively. Red (blue) line is for the case where two electrons occupy the *V*_N_ (N_*i*_) level. The changing color from-red-to-blue region is where the ground-state *V*_N_ and N_*i*_ levels couple to each other.

Figure 4 shows the potential energy manifold during the FP formation. By injecting two electrons and two holes in the CBM and VBM, respectively, the total energy of the system at the initial configuration is increased by twice of the band gap energy (6.81 eV). Here solid lines represent non-crossing regions whereas dashed lines represent the crossing region, as discussed above. The barrier for the FP formation, in the presence of carrier injection, is only 0.56 eV, which is significantly reduced from the 4.79 eV in the ground state. One can qualitatively understand the difference as follows: displacing an N from the defect-free structure increases the total energy of the system. However, the increase is substantially smaller than what would be in the ground state without the excitation, because the significant increase of the depleted N_*i*_ level no longer costs energy and the significant lowering of the occupied *V*_N_ level also helps to offset the energy required to break the bonds and associated strain. After forming the FP, the system will revert to the original defect-free structure, as it only needs to overcome a barrier of 0.47 eV.

We have analyzed the occupied energy level in the ground state during the reverse process back to the defect-free structure. It was found that the character of the occupied defect level is continuously changed from that of the *V*_N_ state to that of the N_*i*_ state. As the occupied N_*i*_ level and the empty *V*_N_ level eventually merge into the VB and CB, respectively, the NRR process assisted by carrier injection is completed. One may classify the transient defect mechanism in terms of a strong electron-phonon coupling, i.e., the significant energy level changes during the FP formation, and the reverse process transforms the initial excitation energy into thermal motion of the atoms.

## Discussion

To see the dominate process in recombination, we compare the radiative recombination time of the excited carrier with the FP formation time. The FP annihilation process is, on the other hand, unimportant here because after the FP formation, the excited electrons already fall in a level lower than the holes (cf. Fig. 3) so that radiative recombination is no longer possible. We can estimate the FP formation time using the rate equation *r* = 3 × *f* exp(−*E*_b_/*k*_B_*T*), where *k*_B_ is the Boltzmann constant, *f* is the optical phonon frequency, 2.0 × 10^13 ^s^−1^ for GaN^25^, *E*_b_ is the formation energy, 0.56 eV, and *T* is the junction temperature during the LED operation, 130 °C^26^. The factor 3 stands for the number of equivalent NRR pathways. The calculated NRR relaxation time is about 160 ns, which is about twice the radiative recombination time of 88 ns^27^. Over this time, about a quarter of the excited electrons undergo the NRR processes.

Note that the magnitude of the band gap is crucial for enabling the NRR process. When the band gap is smaller, the energy required to create two electron-hole pairs through carrier injection is also smaller, for example, in GaAs, it is only 2.84 eV, while the FP formation energy is 4.59 eV^28^. Therefore, no transient FP-formation can take place, even under the carrier injection condition.

## Conclusion

Using density-functional theory calculations, we demonstrate the possible existence of a new NRR mechanism, where the injected high-energy carriers induce a structural instability, namely, the low-barrier transient defect formation, and the associated NRR through strong electron-phonon coupling. While our study is focused on GaN-based LEDs, the theory, as shown in Fig. 1, is general and could be an important limiting factor for other wide-gap semiconductors for the efficiency of their optoelectronic and photovoltaic devices. In particular, it raises the important question whether new mechanism(s) may exist between the widely-accepted defect-based SRH mechanism and the defect-free AR mechanism to account for some of the most difficult but technically important materials issues regarding excited carriers.

## Methods

Our calculations are based on the density functional theory (DFT) with the Heyd–Scuseria–Ernzerhof screened hybrid exchange-correlation functional^29^, as implemented in the VASP code^30^. We use a mixing parameter of 0.3 for the exact exchange, as in previous studies^31^. Projector augmented wave potentials^32^,^33^ are used to represent ion cores. Plane waves with an energy cutoff of 306 eV and 230 eV are used as basis sets for InGaN and GaAs, respectively. We use a periodic supercell that contains 96 and 128 atoms to model the InGaN alloy and GaAs, respectively. Γ point is used for Brillouin zone sum. Atomic structures are relaxed until the residual forces are less than 0.03 eV/Å. To simulate electronic excitation, we perform constrained DFT calculations, in which we remove two electrons from the VB maximum (VBM) and place them at the CB minimum (CBM). Standard reaction-barrier-search algorithms, such as the nudged elastic band method, are not applicable to calculating the energy barrier in the present case, because electron occupation changes during the process. Instead, we generate ten intermediate configurations between the initial and final configurations and then relax all the atoms except for the diffusing N atom and a Ga atom of choice far away from the N.

## Additional Information

**How to cite this article**: Bang, J. *et al.* Carrier-induced transient defect mechanism for non-radiative recombination in InGaN light-emitting devices. *Sci. Rep.* **6**, 24404; doi: 10.1038/srep24404 (2016).
